# ‘I know what I should be feeding my child’: foodways of primary caregivers of Child Support Grant recipients in South Africa

**DOI:** 10.1080/16549716.2021.2014045

**Published:** 2022-02-14

**Authors:** Wanga Zembe-Mkabile, David Sanders, Vundli Ramokolo, Tanya Doherty

**Affiliations:** a Health Systems Research Unit, South African Medical Research Council, Cape Town, South Africa; b Southern African Social Policy Research Institute Cape Town, South Africa; c Archie Mafeje Research Institute, College of Graduate Studies, University of South Africa Pretoria, South Africa; d School of Public Health, University of the Western Cape, Cape Town, South Africa; e Department of Paediatrics and Child Health, Faculty of Health Sciences, University of Cape Town, Rondebosch, South Africa; f HIV Prevention Research Unit, South African Medical Research Council, Cape Town, South Africa; g Gertrude H Sergievsky Center, Vagelos College of Physicians and Surgeons, Columbia University Irving Medical Center, New York, NY, USA; h School of Public Health, University of Witwatersrand, Johannesburg, South Africa

**Keywords:** Child support grant, cash transfers, foodways, food choices, food security, child nutrition

## Abstract

**Background:**

Despite South Africa being an upper middle-income country producing enough food to sustain its population, and having an advanced social welfare system, it has high levels of food insecurity at the household-level. Food insecurity is linked to malnutrition and undernutrition in children. This manuscript addresses gaps in knowledge about food choices and practices of primary caregivers of children in receipt of South Africa’s largest cash transfer programme, the Child Support Grant (CSG).

**Objective:**

The main objective of the study was to explore CSG caregivers’ foodways and the choices they made about what food to buy, where to buy it and for what reasons, in Langa in the Western Cape and Mt Frere in the Eastern Cape.

**Methods:**

We conducted a total of 40 in-depth interviews and 5 focus group discussions with primary caregivers of Child Support Grant recipients younger than 5 years in the Eastern and Western Cape provinces.

**Results:**

Caregivers’ food choices were less influenced by cultural practices and personal preferences, than by financial and physical constraints in terms of what and where to access food. Constraints in food choices were chiefly a consequence of the small amount of the grant, as well as a food environment that only availed foods of a certain quality and type in these low-income communities

**Conclusions:**

The foodways of recipients of social assistance can only be better aligned with nutrition messaging and policy if there are changes in the monetary value of cash transfers, and the food environments of low-income households which determine access to, availability and affordability of nutritious food. Local informal food enterprises play an important role in the food system of CSG recipients and need to be considered in any strategies that seek to reform the food system of low-income communities in South Africa and similar settings

## Background

Despite South Africa being an upper middle-income country producing enough food to sustain its population, and having an advanced social welfare system that transfers cash to more than 17 million people, it has high levels of food insecurity at the household-level. Food security is defined as ‘a situation that exists when all people, at all times, have physical, social and economic access to sufficient, safe and nutritious food that meets their dietary needs and food preferences for an active and healthy life’[1]. Therefore, people are only food secure when food is both available and accessible- food must not only be in the market but people must be able to afford it. Additionally, for an active and healthy life, people need enough food as well as the right balance of fat, protein, carbohydrates and micronutrients. There are thus four dimensions to food security – availability of food, access to food, utilisation of food, and stability of both availability and access [2]. Households are defined as food insecure if they fail in at least one of the dimensions [2].

In Southern Africa food prices are experiencing above-average highs, especially for staple crops like maize. In South Africa food prices have been increasing at alarming rates year on year without respite [3]. This means that poor households are now spending more on basic food items and this reduces people’s ability to access food [3]. Some of the reasons proffered for the increase in food prices include: international food commodity pricing and electricity tariff increases, excessive price increases of retailers, changing consumer shopping trends, higher crude oil prices [4], and climate change related factors such as changing weather patterns and the influence on crop yields [3,5].

**Figure 1.**
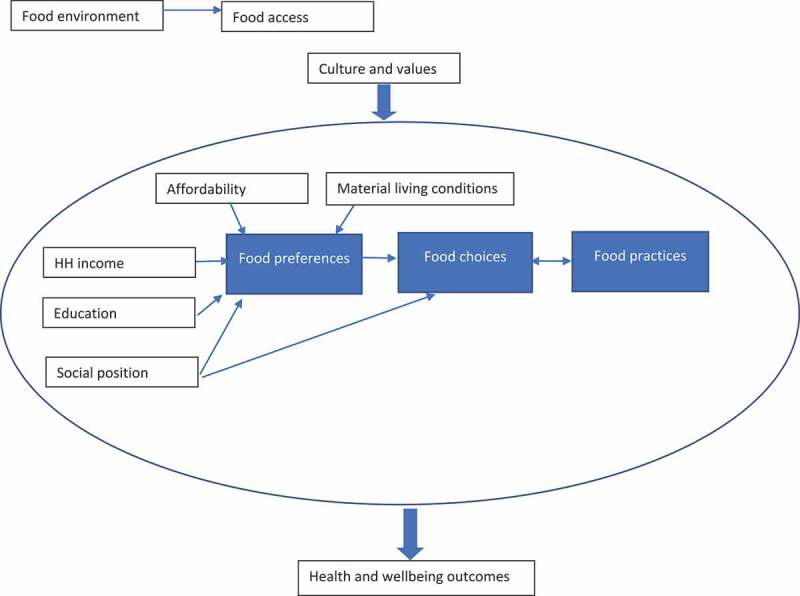
A conceptual framework for understanding how foodways impact on health and wellbeing outcomes.

In the South African context, it is against this background that low-income primary caregivers of young children have to feed their children. However, little is known about their foodways. Foodways is a term denoting the cultural, social, and economic practices related to food production and consumption [6]. It has also been defined as ‘the set of strategies shaping what food people choose as well as how and where they access and consume it’ [7]. In foodways conceptualisation, structural differentiation plays a key role in people’s foodways, with the tastes and preferences of the poor being markedly different from those of the middle and upper classes [8]. According to this conceptualisation of foodways, social class and attendant indicators such as educational level and income drive food preferences [9,10]. However, the correlation between food preferences and social class is not linear or simple. Bourdieu, one of the pioneers of this theory, posits that foodways are shaped by *habitus*, where food preferences are an outcome of a collective schemata or conceptual system for understanding social experiences and perceptions around food. These experiences and perceptions are a result of an individual having internalised childhood experiences and material living conditions within the context of family and other exposures such as schooling [8]. Thus, one’s social position leads to a practical embodiment of habitus in how they eat, drink and carry themselves in society [8]. Therefore, according to this theory, people’s choices about what to eat, how to prepare and eat food, and when, where and with whom to eat, are not a simple matter of whether they have money or not in the present, as those preferences would have been shaped from birth by the context they grew up in, even as the context itself may have been shaped by socio-economic status or social position and class. As such, a simple conceptual framework for foodways (Figure 1) posits context, culture and values as mainly responsible for influencing food preferences and food practices, and these factors in turn ultimately influence the choices that people make about what and where they access food.

Understanding the foodways of food insecure primary caregivers of children is important because food insecurity is linked to various adverse health outcomes for children such as malnutrition, specifically undernutrition [11]. Undernutrition has negative ripple effects on timing of entry into school, educational attainment and economic productivity, ultimately resulting in intergenerational poverty effects, it also triples the risk of having stunted children and of suffering from obesity and resultant chronic illnesses in adulthood [12]. Undernutrition is linked to other poverty-related factors besides food insecurity, such as poor sanitation, socio-economic status, hygiene, food preparation methods, and maternal education [13]. The 2017 South African General Household Survey (GHS) reports stunting for children under five at 27%, and this places the country at 67th highest out of 136 countries in the global ranking of stunting prevalence [14].

Along with high levels of undernutrition, a sharp increase in obesity and overweight has been observed in South Africa, which has led to an increase in the prevalence of non-communicable diseases (NCDs) [6]. Among children, overweight rates were recorded at 13% in 2017 [7]. This means that the country is facing a double burden of malnutrition with high rates of stunting on the one hand and rising levels of obesity and overweight on the other. Poverty, food insecurity and precarious living conditions are the biggest drivers of the observed high malnutrition rates, all occurring in the context of a rapidly changing food environment characterised by a shift in diet from traditional diets, towards a more ‘western’ diet consisting of highly processed and cheaply accessible foods [15].

Within this context, social protection, especially in the form of cash transfers, is regarded to be an important instrument in the fight against child poverty and poor child health and nutritional outcomes. Cash transfers in the hands of women are hypothesised to enable greater food access, affordability and choice [16]. Established in 1998, the Child Support Grant (CSG) is the largest cash transfer programme in South Africa and the Continent, transferring money to more than 12 million children from low-income households, which translates to about 80% reach. The value of the grant is currently set at R460 per recipient per month (about US$28^1^^1^R330 at the time of the study (US$21) in 2015.). It is unconditional and non-contributory, but it is means-tested with an income threshold set at 10 times the value of the grant.

Given this background, and especially in the current context of the COVID-19 pandemic, it is important to understand how primary caregivers of children receiving South Africa’s largest child cash transfer, the CSG, approach the challenge of food insecurity and what informs the different choices, strategies and access channels they use to feed their children. A number of surveys have been conducted on food insecurity, hunger and malnutrition but there is less detail on people’s experiences of coping with these conditions from a foodways perspective. This manuscript reports findings from a qualitative study that explored CSG caregivers’ foodways and the choices they made about what food to buy, where to buy it and for what reasons, in South Africa. The study sought to understand how low-income populations experience food and foodways in the context of poverty, food insecurity and social protection. The manuscript makes an important contribution to the current evidence base on foodways of low-income individuals and households, as it is the first qualitative paper to look at foodways of primary caregivers of recipients of social assistance in a low-and-middle income country setting.

## Methods

This qualitative study draws its data from a larger study conducted to provide an in-depth examination of the CSG and its role in child wellbeing and food security in an urban township in Langa, Western Cape Province and in a rural setting in Mt Frere, Eastern Cape Province. The methods of the study have been reported in a previous publication [17].

For the component of the study reported in this manuscript, this exploratory qualitative sub-study focused on mothers and caregivers’ foodways -what, where, why and how caregivers accessed food for their children and households in an urban township in Langa, Western Cape Province and in a rural setting in Mt Frere, Eastern Cape Province.

While the data were collected in 2015 the findings remain relevant as no major changes have occurred in the eligibility criteria, delivery system, and benefit level of the CSG. The national take-up rate of the CSG has remained similar at +-80%, as have the eligibility criteria, the mode of delivery (cash or electronic payments), and the benefit level which has not increased drastically in real terms, having not kept up with inflation in the last few years.

### Study setting

Mt Frere is a small rural town in the Eastern Cape Province. It forms part of the apartheid-era Former Homelands established under the Bantu Homelands Citizenship Act of 1970. Homeland areas are characterised by high levels of poverty, lack of resources, poor administration systems, and poor access to services; all a legacy of the apartheid era which isolated these areas. In the South African Indices of Multiple Deprivation (SAIMD 2001; 2011) Mount Frere repeatedly comes up as falling under a ward with the highest levels of deprivation in the country [18]. This little rural town has been profiled more than once as an area with the highest rates of child malnutrition, hunger and poverty [19].

In Mount Frere, rural villages mainly have village trading stores which sell everything from groceries, to clothing and furniture; and grocery stores that mainly sell household staples. In town, both formal and informal food enterprises and large retail chain stores stand side by side.

Langa township in the Western Cape is home to 52,401 residents [20]. It is the oldest township in Cape Town, first established in 1927 as part of the Urban Areas Act of 1923 which sought to segregate racial groups in South Africa. Thus, it has a lot of history, and has seen various changes in its 90 years of existence, including the expansion of large informal settlements, an established (though small) low middle-class, and a strong informal sector, which in recent years has begun to compete with formal large retail chain stores recently introduced in the township. Even though Langa township is in an urban setting, its spatial and economic exclusion from wealthier and more central parts of Cape Town make it a marginalised area suffering some of the same issues faced by peri-urban and rural settings in South Africa, such as high levels of poverty and deprivation, poor living conditions and poor access to quality health services, education and employment opportunities.

The food system in Langa is characterised by a mix of formal and informal food enterprises and large retail chain stores. Informal food enterprises include small grocery stores colloquially known as ‘Spaza’ shops, fruit and vegetable stalls, and cooked meat stalls.

### Sampling frame

The sample for this study was drawn from the Prospective Urban Rural Epidemiology (PURE) cohort study focusing on non-communicable diseases in 18 countries [17,21]. At the time of the study reported herein, the cohort was following approximately 2000 individuals, and from this number a total of 40 mothers or primary caregivers in receipt of the CSG for children under 5 years in the two sites were recruited for in-depth interviews. We focused on children younger than 5 years because of the importance of the first 5 years in a child’s life in determining nutritional outcomes.

Participants from the cohort were identified by a research assistant through a participant spreadsheet which contained details about the households enrolled in the study. From the spreadsheet the research assistant identified households that met our eligibility criteria, that is, a household with a primary caregiver of a child under 5 residing in Langa or Mount Frere. The households that met the eligibility criteria were each called to inform them about the study and to ask for permission to visit the primary caregiver to talk about the study. Primary caregivers who agreed to have an initial visit to their homes were visited and after explaining the purpose of the study through reading the information sheet, those who consented to participation in the study were interviewed. A profile of the study participants is presented in Table 1. The information presented relates to CSG receipt status, average household size, educational levels, and employment in each site. Participants ranged in age from 18–70 years, and with 6 grandmothers interviewed as primary caregivers of the index children. In terms of marital status, the rural site, Mount Frere had more married respondents than the rural site, Langa. Employment was low in both sites, with no participants in employment in Mount Frere, and only 3 participants reporting formal employment in Langa. Educational levels did not exceed secondary school in either of the two sites.

**Table 1.** Profile of participants included in individual interviews (n = 40)

	Mount Frere (n = 20)	Langa (n = 20)
Household size range	2–6	2–14
Average household size	4.3	5.6
Age range of caregivers	18–70	18–65
Marital status Single Married Widowed	3 15 2	15 4 1
CSG caregivers in formal employment	0	3
Caregivers who have not completed secondary school	17	6
Caregivers who have completed secondary school	3	14
Relationship of caregiver to child	Mother (n = 13) Grandmother (n = 7)	Mother (n = 18) Grandmother (n = 2)

Five focus group discussions (FGDs) with approximately 8 members per group were conducted in addition to the in-depth interviews. All group discussions had female participants except for one group which had 1 male participant in the Langa site. The groups were mainly made up of older participants with the age range of group participants being 25–70 years old. A community recruiter in each study site identified participants for the FGDs. The recruiters’ brief was to recruit community members over 18 years of age with children, thus the final make-up of the groups in terms of age and sex was not influenced by the study. The main objective of the focus group discussions was to gain community level views and perspectives on how primary caregivers secured food for their children, and the role of the CSG in food security and children’s diets. Group discussions took place in community halls in the two study sites.

### Data collection and analysis

Interview topic guides were developed by the lead author along with the study co-investigators prior to the start of the study. The final drafts of the topic guides were translated into isiXhosa, and piloted by the lead author and VR in Langa township. Several changes were made to the topic guides after piloting. Following piloting, in 2015 the lead author, a Black African isiXhosa speaking female with a rural upbringing as well as exposure to urban township life, together with VR, also a Black African female with an urban township upbringing, conducted all in-depth qualitative interviews and focus group discussions in the two sites. Both interviewers have had training and extensive previous experience conducting qualitative interviews Individual interviews took place in participant homes, and FGDs took place in local community halls. Participants for in-depth interviews were different from FGD participants and participation in both by the same participant was not allowed, as we wanted, as far as possible, to be able to keep individual experiences and perspectives, different from community level views and perspectives. The interviews were conducted in the main language spoken in both sites, isiXhosa. Interviews were conducted until data saturation was achieved.

Interviews were mainly conducted with only the primary caregiver present, and only had more than one person sitting in in an interview if they were also directly involved in the raising of the index child. In many instances the additional person would be grandmother of the child who would be playing a key role in raising the index child. In many cases, as is the norm in Black African communities in South Africa, family members, neighbours and friends would go in and out of the participant home wanting to speak to the primary caregiver. In such cases the interviewers would wait for the conversation or interaction between the participant and the person coming to speak to them to finish, and for that person to leave the room, before resuming the interview. In many such cases, the participant would inform the person that they had visitors and for them to come back later and so the interruption would not last long.

During each interview, the researchers wrote field notes to capture the context, home environment and non-verbal communication. In the early days of data collection, WZM and VR discussed and compared notes after each interview they conducted together and these were used to guide further interviews where appropriate. The field notes were also used to write summaries of each case/interview for sharing with co-PIs, and were used in analysis workshops to understand the context of particular cases of interest.

Graneheim et al’s [22] manifest and latent thematic content analysis methods informed the process of analysing the data. Transcription and translation of audio tapes were conducted by independent transcribers. The lead author performed the initial checking of every transcript for accuracy, especially of translation from isiXhosa to English, and where there were inconsistencies or errors these were sent back to independent transcribers to re-transcribe and translate.

In-depth interviews were analysed first before FGD transcripts and the coding was initially kept separate, but during the process of analysing FGD data, similar themes were noted and the coding was merged. The lead author developed a list of all the interviews and transcripts and captured these in Microsoft Excel and manually copied and pasted passages of text from Microsoft Word to categorise the data. No qualitative data management software was used. Even though the lead author undertook coding of the data, all co-authors were heavily involved in the analysis and interpretation of data. They read summaries of interviews and read through some of the transcripts as part of the data validation process of emerging themes. Initial codes were grouped together into categories that were then further transformed into major themes. Several meetings were held to discuss the data, including two separate 2-day data analysis workshops where consensus on major themes was established and manuscript outlines were developed.

Transcripts were not shared with the participants. However, we were guided by ethics protocols in how interviewees were encouraged to raise questions throughout the interview and the interviewers made sure to reflect back comments to participants to ensure accurate capturing of data and interpretation.

In addition to the thematic analysis described above, for this manuscript, we have additionally utilised a qualitative case study approach to provide greater detail on some of the participants and the experiences and views they shared. The case studies are made up of participants from the same sample of primary caregivers who consented to the study and were interviewed in the same way. We have used the case study format to present a more in-depth description in order to highlight their stories. The case study approach in the context of qualitative research methods entails analysing and describing a study participant individually (their activities, life situation, life history etc) in detail [23]. In this manuscript we present two case studies of individual participants and their life situations, along with thematic results from the remaining participants.

## Results

In analysing data for the substudy reported in this manuscript we focused on caregivers’ decision-making processes around spending the CSG on food. Specifically, caregivers spoke about their motivations around the choices they made concerning food -what food they bought with the CSG money, how much, where they bought it, and their reasons for buying it.

Major themes related to the topic of food choices centred around i) price, convenience, availability, and clever marketing; ii) food storage concerns, and iii) the consumption of snacks.

The first theme related to several aspects of pricing, convenience, availability and marketing. Convenience was experienced in terms of food preparation and how long it took to prepare and its taste. It also focused on ease of availability of some foods and the ways in which food was advertised and marketed as being nutritious, tasty and healthy influenced primary caregivers’ food choices.

The second theme related to the availability of food storage, especially refrigeration, and how this shaped and constrained primary caregivers’ choices about what food to buy.

The consumption of snacks theme contained data about the primary caregivers’ use of snacks as a large proportion of their children’s diets, and the motivations behind this practice.

### Caregivers’ choices about what food to buy are influenced by price, convenience and clever marketing

As recipients of the CSG, valued at R330^2^^2^USD 23.29. at the time of the study, most participants in our study did not have a lot of money to spare, and therefore food pricing was a major factor in primary caregivers deciding what food items to buy. Often primary caregivers tried to buy something that was both cheap and filling to maximise and stretch the money they had to last until the next pay date. One mother in Langa explained her choice of feeding her child a sugar laden refined instant breakfast porridge as it was both low in price and filling. Many other caregivers who were interviewed followed the same logic in their food choices for their children.

*“Morvite only costs R15 per packet and you get a lot when you mix just a little bit of it and the child stays full for longer” (CSG FGD2, Langa)*

*The porridge that I prefer my child to eat is Oats, Weetbix and New Life [instant porridge], New Life is a new porridge. I like it [New Life porridge] because it tastes nice, [and] when you make it as a milkshake for your child, you don’t have to make them a sandwich, you just give him the milkshake and they will be full the whole day (CSG recipient, Langa)*

Some caregivers believed that popular foods consumed by children in low-income households like instant noodles were unhealthy, but were confused by the advertising on TV which showed different meat flavours, which suggested that the meals were nutritious and were ultimately persuaded by the convenience (quick preparation), the low price and popularity of the product to make this a staple in their children’s diets.

*“No, I can’t speak highly of [instant noodles], shame.[but]* t*hey are advertised on TV and they show different flavours, there are beef, chicken and stirfry ones, you can make them for your child everyday and you will never hear a complaint about hunger after that” (CSG recipient, Langa)*

Caregivers conceptualised a healthy diet as comprising vegetables and fruit, meat, dairy and starch. However, many felt that such a diverse diet was beyond their means, and attested to mainly feeding their children starchy food as it was filling.

*“The quality of food isn’t right because a child would end-up eating what is cooked at home, whatever is cooked the child will be given that, not the food that is meant for a child. The food has no vitamins, things like fruit and vegetables that a child requires aren’t available. The child ends up eating what is on offer at home because the grant only pays for school fees and transport, that’s it. It would be better if SASSA was giving people money and then in addition vouchers strictly for food. Otherwise a child ends up eating porridge that is meant for the whole family everyday of the week, that is definitely not healthy for a child” (CSG FGD, Langa)*

However, there were caregivers who still tried to feed their children what they considered to be healthy despite having little money

“*I know what I should be feeding my child, because they tell us at the clinic, children must eat vegetables, they must have meat, they need fruit, they need yoghurt and cheese, they should drink milk once a day, but that is a luxury. I try to get them vegetables by buying [frozen] mixed veg because its a bit cheaper, and I add a bit of it to the sishebo, or the gravy sometimes, when I get their money I try and get them fruit and some snacks that they like, but that’s only on that [first] week they eat like that, then [its back to the usual].”*(CSG recipient, Langa)

*“For instance if a child eats soft rice, mixed with potatoes, a little oil and a vegetable like spinach, a child will eat and be satisfied from this” (CSG recipient*, Langa)

The diet of the majority of the children in the study included a cultural staple in African communities in South Africa, called *umvubo*, which is a mixture of fine-grain maize meal (*Uphuthu*) and sour milk (*Amasi*). Even though caregivers talked about feeding their children this staple because it is a popular (and preferred) dish with children and adults alike, they also mentioned that they chose it because it was quick and easy to prepare, and it was cheap. Thus, convenience and affordability, even with a preferred meal, remained important in caregivers’ decision-making about what to feed their children. Other culturally preferred dishes such as *Umngqusho* (samp and beans) are not regularly consumed because they are expensive, both in terms of price and cooking time.

The lack of diverse diets was clear in the food hampers that were popular with many caregivers in both sites. Many caregivers in both Mount Frere and Langa reported that they bought their food in bulk through food hampers that were mainly sold in *spaza*^3^^3^Small local shops selling grocery items. shops owned by foreign nationals. The main reason for buying the hampers which were popularly known as ‘combos’, was that they were cheap and yet contained all the major staples. The popularity of the hampers reflects the important role that small [informal] traders (spaza shop owners) play in the food system – their hampers were often made up of products of inferior quality which enabled them to keep prices down – some caregivers were aware of the inferior quality of the products but felt that they had no choice but to go with what is cheap. Most of the respondents bought their staples from Spaza shops with a clear preference for Somalian-owned shops; and opted to buy smaller items like soup, tinned food, margarine, baby foods (including Instant Porridge and Morvite) from large chain supermarkets.

*“We buy hampers … we buy it from the Somalians. it has mealie meal, sugar, flour, rice, oil, samp and beans.” [it costs] only R300 and something (CSG recipient, Langa)*

*“What I do is I buy a hamper from the foreigners for R350 …. that at least is a good buy ….The hamper has 10kg rice, maize meal 10kg, 10kg flour, 10kg sugar, and 2L cooking oil and Cremora milk powder ………. They at least last me a while … ” (CSG recipient, Langa)*

Hampers sold in the villages of Mount Frere were much more expensive than those in sold in Langa, and that was likely due to the remoteness of the areas which meant shop owners incurred more costs in transporting goods. However, the cost was still less than individually buying the food items from local retailers in town.

*“I buy … I buy those [grocery] hampers because you get a hamper for about R530. From the hamper you get 10kg of Sugar; 10kg Flour; 10 kg Mealie meal; 10 kg of Rice, 5kg of the yellow mealie meal and two litre fish [cooking] oil, that is all. Then I would need to add more money to get izishebo (side dishes)” (CSG recipient, Mt Frere)*

Caregivers demonstrated an astute awareness of how some shop owners used the grant to manipulate prices to maximise profits.

*“All the shops in town raise their prices on the day when we get the grant –some of them you can even see that they have scratched out the old [lower] price [on the product] and replaced it with a higher price. They do this for the first 3 days after we get paid, then they lower the prices again …… [as a result] no matter how hungry I am I try to wait until those first 3 days pass before I do my shopping ……. it is not fair because this grant money is already so small, its not like we are teachers or nurses who earn big salaries, the shop owners should raise the prices on the 20^th^ of the month when people with a lot of money like teachers get paid, not when its poor people like us ….” (CSG FGD 1, Mt Frere)*

### The role of food storage concerns in determining food choices

Some caregivers’ choice of what to buy and feed their children was constrained by food storage challenges. In both the informal parts of Langa and rural Mt Frere a few households did not have refrigerators or if they had them, they could not operate them due to lack of electricity and this meant that they could not buy perishables in bulk. The lack of a fridge was a direct result of [lack of] affordability, as well as unstable access to electricity in some of the areas.

*“I buy meat for that moment if I have money, if I don’t have money then I don’t have money, [otherwise] I don’t buy meat because I don’t have a fridge …. it is not on the budget” (CSG recipient, Langa)*

*“I do have a fridge, but I do not have electricity so I cannot use my fridge, so I would need to take the food that needs a fridge to a friend who has one ….then from time to time I go and get those few pieces of meat and cook for the children ….but I keep the sour milk here ….in the afternoon if its hot, I make them sour milk and mphokoqo (mealie meal dish), and sometimes we eat the sour milk meal again for supper ….”(CSG recipient, Mt Frere)*

### Consumption of snacks to stave off hunger and keep children happy

In most households we visited, children frequently consumed nutrient-poor, energy-dense sugary snacks such as cheap sweets (lollipops), biscuits, chips, sweetened yoghurt and cordial/squash drinks. In many of the households that could barely afford three meals a day, children were still able to have regular helpings of snacks. Many caregivers rationalised this as a small and affordable way to keep their children happy, and some suggested that snacks were an easy and cheap way to keep hunger at bay.

*“ … he likes biscuits and chips … [he gets them] from people. If I have money I buy [the] snacks* … ” (CSG Recipient, Langa)

“ *And then I also buy chips … things that will make them happy; [such as] yoghurts …… I buy a bag … of fifty” [chips a month]. (CSG recipient, Langa)*

*“When I give them a lollipop and chips and juice or rooibos during the day at least it keeps them [preoccupied] until I can cook for them later on” (CSG recipient, Langa)*

### Case studies

The following two case studies illustrate how two families navigate foodways to provide sufficient food for their families.

#### Mt Frere: case study 1

This was a family of 5 – mother (41), father (37), and three children between ages 15 months to 9 years. The primary caregiver was the biological mother of the three children who were CSG recipients. She was unemployed and survived on 3 CSGs (total of R990 a month, US$60.93). The father was a carpenter and builder with unstable employment but even when he did earn money, he rarely contributed to the household income.

The mother spent most of the CSG money on groceries. Accessing food in this rural setting came with high transport costs. The family lived about 15kms from town and paid R10 each way for herself and then R4 for each big grocery item (e.g 10 kg mealie-meal, sugar). As a result, she preferred to buy a grocery hamper at a nearby village store for R530 (US$32.62), and then bought *izishebo* in town at Shoprite or Boxer or Spar (Soli’s)

“*No I do not buy my groceries in town, I only buy zishebo in town …*. I *usually buy 5kg chicken; a bag of potatoes; 5l sour milk and then the money runs out once I buy those things. The trouble is with loading everything on a taxi, they will charge for each of the big items. The only items you do not pay for are those that are in plastic bags …*.”

The caregiver’s choices about what food to buy for her children and household were mainly influenced by affordability, as well as energy density (to ensure satiety for hours at a time). As such, she mainly fed her children cheap starchy food, and frequently ran out of food before the end of the month

“*Yesterday, early morning she ate her mealie porridge when she woke up … for lunch she ate her mealie pap and soup ……… for dinner she ate sour milk with umphokoqo (phuthu –fine grain mealie-meal) ………. No, there is nothing she eats [between meals], she likes drinking water sometimes … ”*

#### Langa case study 2

This was a family of 8 – mother (18) with two children aged 8 months and 2 years, older sister (27), older brother (24), younger brother (14), the 18 year old caregiver’s mother (54), and 2 additional children under 5. The 18-year-old mother was unemployed and in grade 10. The family survived on 3 CSGs (at the time this was a total of R990/US$60.93 a month), occasional earnings from the 24 year old brother’s ‘piece jobs’, and grandmother’s proceeds from her veggie stall – ”*it’s not something that brings in a lot of money, some days it just makes R20”*. The young caregiver’s mother was in charge of the household income. The family rented a 2-room shack for R350 (US$22.78) a month and spent about R500 (US$32.07) a month on groceries. In this household school related expenses – transport, uniforms, and fees -were prioritised above food and other needs. The family diet, including food eaten by the children, was mostly starchy, with little diversity.

*“They [children] eat whatever is in front of them. Porridge, rice, potatoes as well. Milk no, they only get it when I have money, then I’ll buy them then … right now they drink Rooibos [tea]”(Grandmother, CSG recipient, Langa)*.

Groceries were purchased from a nearby Spaza shop owned by Somalians and the family only went to Shoprite USave for ‘small items’ – soup powder, margarine, morvite, washing powder and toiletries.

*“What I do is I buy a hamper from the foreigners for R350 …. that at least is a good buy ….The hamper has 10kg rice, maize meal 10kg, 10kg flour, 10kg sugar, and 2L cooking oil and Cremora milk powder ………. They at least last me a while … ”*

The family appreciated the CSG, but acknowledged that it was not enough to meet the nutritional needs of its beneficiaries, let alone the household’s. Similar to other CSG recipients, this family was trapped in a vicious cycle of debt – borrowing food *and* money to get by every month.

“*It [the grant] helps, because you can hear of the things I’ve mentioned, I was not going to be able to get those things without it, because their sister is not working, no one here works*”

“*You see now I just paid this [Spaza shop debt and school fees] money, its difficult for the [kids] and me to go home [to the Eastern Cape] at the end of the year, we no longer go home, I made peace with that …*.”

“*I borrow [money], yes ….just like this girl [who just called] she is one of the people that help me out with money … ”*

Social capital was important in getting this family through difficult times that required increased spending such as the December period where many families usually spend money buying lavish groceries to celebrate Christmas. For this family, the CSG money was usually finished by the 20^th^ of December and buying special groceries for Christmas was a luxury they could not afford, but the primary caregiver was grateful to her neighbour who always invited her and her family over to her house for a festive meal on Christmas Day.

*“[On Christmas day] they help us here, the children [neighbour] make a party, you see especially this one [neighbour] she can cook really well, she cooks here at Mjoli’s and dishes out for us and so we are able to eat well on Christmas day*”.

## Discussion

This qualitative research sought to understand the foodways and choices of caregivers who are in receipt of the CSG on behalf of their children. To our knowledge, this is the first qualitative study to address the topic of foodways in the context of social protection in a low-and-middle income setting.

The findings show that caregivers’ choices about what to feed their children were less influenced by cultural practices and personal preferences, as traditionally conceptualised in foodways theory, than by financial and physical constraints in terms of what and where to access food. Constraints in food choices were chiefly a consequence of the small amount of the grant, as well as a food environment that only availed foods of a certain quality and type in the low-income communities. Thus the findings add greater nuance to traditional conceptualisations of foodways as being primarily shaped in childhood and largely remaining static in adulthood [8]. In our study primary caregivers received nutrition education from health facilities about what to feed their children and they were prepared and willing to change their foodways to conform to what they had been taught, however, it was cost and access that ultimately determined what they fed their children.

Our findings further showed that informal enterprises such as spaza shops played an important role in the food system because they were cheap and convenient places to purchase groceries in bulk. The findings of this manuscript further revealed specific practical factors, such as food storage and preparation concerns, as determining the food choices of low-income families in our study.

Similar to findings of the larger cohort study [21] from which participants for our study were sampled, which showed that cohort participants had high carbohydrate intake [21], our findings also show that the diets of the children and primary caregivers in our study were mainly starch-based, with a high consumption of snacks. The finding on snacking adds an important dimension to the body of work that exists on parenting and child snacking [24], as consumption of nutrient-poor snacks, ordinarily associated with poor food choices, was shown to be a rational choice in our study, as caregivers who had little to give to their children ‘to make them happy’, were able to use the snacks both to bring joy, as well as to pacify hunger.

The foodways of low-income households in South Africa have been explored before, albeit without the focus on the role of social protection in shaping such practices. Kroll [25] explored the foodways of poor households in South Africa through secondary analysis of national surveys. His findings showed that poverty does not make poor people passive consumers in the food system, rather they make rational, deliberate decisions about what food to eat, where to purchase it and how to deal with constraints presented to them by the food system [25]. This is supported by our own findings in this manuscript which showed low-income caregivers to be deliberate and rational in the food choices they made for themselves and their children, even in instances where those choices seemed to be negative or nonsensical, as with the choice to allow children to frequently consume nutrient-poor snacks.

Our participants were also deliberate in their choices about where to buy what food, with the majority preferring to purchase bulky food items (food hampers) at local spaza shops, or rural stores, and smaller items regarded as ‘*izishebo*’ or starch accompaniments being purchased in retail supermarkets. Charman et al [26] reported in their study on purchasing patterns of residents of Phillipi, a local township in Cape Town, that the preference for food hampers purchased in spaza shops was logical as the spaza shops often sold the hampers at cheaper prices and were located nearer to homes, and thus residents did not have to pay to transport big staples to their homes. An interesting finding about the role of informal enterprises is that, contrary to expectations, the move to build shopping malls with big retail supermarkets in township areas does not seem to be displacing these small local enterprises. Charman [26] and Petersen [27] similarly report in their studies on local enterprises in townships, that these have not been impacted on by the introduction of shopping malls and chain retail supermarkets in these areas. They argue that this makes sense as consumers use each type of enterprise to meet different and specific needs, as evident in our data.

Similar to our findings, Temple and Steyn [28] found in their research on the consumer patterns of the poor in South Africa, that price and energy density were important drivers of food choices, and further found that food prices constrained choice about what to buy and where to buy it. The authors’ research showed that in 2011 a healthy diet cost nearly 70% more than an unhealthy one, limiting poor people’s ability to choose healthy foods [28]. Researchers in the US have similarly reported that ‘cost, not lack of knowledge or physical distance, is the primary barrier to healthy food access, and that low-income people employ a wide variety of strategies to obtain the foods they prefer at prices they can afford’ [29]. In our study caregivers were clear about their knowledge of what constitutes a healthy diet, and were equally clear about their inability to afford such. It is thus noteworthy that even though high food prices impact on the poor the most, and along with competing needs, contribute to the erosion and dilution of social grants in the South African context [30], there continues to be no attempt to review or regulate the pricing of food [25], nor to link the value of the CSG to food price movements as previously recommended [17,30,31].

Finally, our findings on food storage concerns impacting on the food choices of low-income caregivers is supported by Kroll [25]’s assertion that cookware, appliances and other material culture used by people in foodways make as much of a statement about poverty and wealth as the choices people make about what to eat, where to buy and with whom to eat it.

## Conclusion

Caregivers know what to feed their children; the foodways of caregivers of CSG recipients are rational and are mainly driven by food affordability, accessibility and availability. Caregivers of CSG recipients and their children mainly subsist on energy-dense starchy food with little diversity, not because they are not aware of the benefits of a diverse diet consisting of all 5 main food groups, but because they are constrained by the little money they have. The evidence presented in this manuscript shows that beyond values and culture, in contexts of poverty, food and foodways take on a particularly complex hue as access to and choices about what food to eat are informed by more than culture and values. They are rather critically linked to access, structural poverty, and the ways in which these factors shape the food systems of low-income individuals and households.

Foodways of recipients of social assistance cannot align with nutrition messaging and policy without a change in the value of cash transfers, and the food environments of the poor which determine access to, and availability and affordability of nutritious foods.

Local informal food enterprises or spaza shops play an important role in the food system of CSG recipients and need to be considered in any plans to reform the food system that shapes the eating patterns of low-income communities.

## Paper context

Traditional conceptualizations of the foodways emphasise the role of culture and food preferences as mainly influencing food choices and practices. However, our study shows that affordability, availability and accessibility were the main factors influencing mothers’ choices and practices around food.The manuscript makes an important contribution to the current evidence base on foodways as this is the first study to look at foodways in the context of social protection within a low-and-middle income country setting.

## References

[1] FAO, editor Declaration on World Food Security. World Food Summit. Rome; 1996.

[2] Ziegler J, Golay C, Mahon C, et al. The fight for the right to food: lessons learned the graduate. London: Palgrave Macmillan; 2011.

[3] Van Wyk RB, Dlamini CS. The impact of food prices on the welfare of households in South Africa. S Afr J Econ Manage Sci. 2018;21. DOI:10.4102/sajems.v21i1.1979

[4] Sikuka W. Rising food price inflation in South Africa causes concern for consumers. Voluntary report – Voluntary - Public distribution. Global Agricultural Information Network; 2021; 2021 May 09. Contract No.: SF2021-0031.

[5] FAO. Soaring food prices and food security. FAO Advisory Committee on Paper and Wood Products 10 June 2008; Bakubung, South Africa: FAO; 2008.

[6] James G. Rice, R. M. H.B. Sigurjónsdóttir. Sigurjónsdóttir, Serving neglect: Foodways in child protection cases. Food and Foodways, 2019:27(4);253-272.

[7] Kroll F, Foodways of the poor in South Africa: How value-chain consolidation, poverty & cultures of consumption feed each other. 2016, Institute for Poverty, Land and Agrarian Studies (PLAAS); Centre of Excellence in Food Security: Cape Town.

[8] Debevec L, Tivadar B. Making connections through foodways: contemporary issues in anthropological and sociological studies of food. Anthropol Notebooks. 2006;12:5–11.

[9] Tivadar B, Luthar B. Food, ethics and aesthetics. Appetite. 2005;44:215–233. 15808896 10.1016/j.appet.2004.10.002

[10] Warde A, Martens L. Eating out. Cambridge: Cambridge University Press; 2000.

[11] Khatun W, Alam A, Rasheed S, et al. Exploring the intergenerational effects of undernutrition: association of maternal height with neonatal, infant and under-five mortality in Bangladesh. BMJ Glob Health. 2018;3:e000881. 10.1136/bmjgh-2018-000881 PMC6254740 30498585

[12] Steyn NP, Bradshaw D, Norman R. The medical research council technical report on dietary changes and the health transition in South Africa: implications for health policy. Cape Town: Medical Research Council; 2006.

[13] Cumming O, Cairncross S. Can water, sanitation and hygiene help eliminate stunting? Current evidence and policy implications. Matern Child Nutr. 2016;12:91–105. 10.1111/mcn.12258 PMC5084825 27187910

[14] StatsSA. General household survey 2017. Pretoria: Statistics South Africa; 2018.

[15] Thow AM, David S, Eliza D, et al. Regional trade and the nutrition transition: opportunities to strengthen NCD prevention policy in the Southern African Development Community. Glob Health Action. 2015;8:28338. 26205364 10.3402/gha.v8.28338 PMC4513184

[16] Bastagli F, Hagen-Zanker J, Harman L, et al. Cash transfers: what does the evidence say? A rigorous review of programme impact and of the role of design and implementation features. London: Overseas Development Institute; 2016.

[17] Zembe-Mkabile W, Surender R, Sanders D, et al. “To be a woman is to make a plan”: mothers’ experiences of the Child Support Grant in supporting children’s diets and nutrition in South Africa. BMJ Open. 2018;8:e019376. 10.1136/bmjopen-2017-019376 PMC5922468 29691242

[18] Zembe-Mkabile W, Noble M, Wright G, et al. Evaluation of poverty and relative deprivation at small area level for the Eastern Cape Province, Report produced by SASPRI for the Office of the Premier, Eastern Cape Provincial Government. Cape Town: Southern African Social Policy Research Institute (SASPRI); 2014.

[19] Telzak SC. Trouble ahead, trouble behind: perceptions of social mobility and economic inequality in Mount Frere, Eastern Cape and Newcastle, KwaZulu-Natal; 2014. Available from: Accessed 30 September 2020. http://www.cssr.uct.ac.za/sites/default/files/image_tool/images/256/files/WP%20326.pdf

[20] StatSA. Census 2011 census in brief. Pretoria: Statistics South Africa; 2012.

[21] Dehghan M, Mente A, Zhang X, et al. Associations of fats and carbohydrate intake with cardiovascular disease and mortality in 18 countries from five continents (PURE): a prospective cohort study. Lancet. 2017;390:2050–2062. 28864332 10.1016/S0140-6736(17)32252-3

[22] Graneheim U, Lundman B. Qualitative content analysis in nursing research: concepts, procedures and measures to achieve trustworthiness. Nurse Educ Today. 2004;24:105–112. 14769454 10.1016/j.nedt.2003.10.001

[23] Verschuren P. Case study as a research strategy: some ambiguities and opportunities. Int J Soc Res Methodol. 2003;6:121.

[24] Blaine RE, Kachurak A, Davison KK, et al. Food parenting and child snacking: a systematic review. Int J Behav Nutr Phys Act. 2017;14:146. 29096640 10.1186/s12966-017-0593-9 PMC5668962

[25] Kroll F. Foodways of the poor in South Africa: how value-chain consolidation, poverty & cultures of consumption feed each other. Cape Town: Institute for Poverty, Land and Agrarian Studies (PLAAS), Centre of Excellence in Food Security; 2016.

[26] Charman A, Bacq S, Brown K. Spatial determinants of formal retailers’ impact on informal microenterprises in the township context: a case study of Philippi East, Cape Town. Cape Town: DST-NRF Centre of Excellence in Food Security; 2019 Feb. 2019. Report No: Research Report 002.

[27] Petersen L, Thorogood C, Charman A, et al. What price cheap goods? Survivalists, informalists and competition in the township retail grocery trade. Cape Town: Institute for Poverty, Land and Agrarian Studies, University of the Western Cape; 2019.

[28] Temple NJS, Steyn NP. The cost of a healthy diet: a South African perspective. Nutrition. 2011;27:505–508. 21074973 10.1016/j.nut.2010.09.005

[29] Alkon AH, Block D, Moore K, et al. Foodways of the urban poor. Geoforum. 2014;54:119.

[30] Devereux S, Waidler J. Why does malnutrition persist in South Africa despite social grants? South Africa: Centre of Excellence in Food Security; 2017.

[31] Zembe-Mkabile W, Surender R, Sanders D, et al. The experience of cash transfers in alleviating childhood poverty in South Africa: mothers’ experiences of the Child Support Grant. Glob Public Health. 2015;10:834–851. 25685927 10.1080/17441692.2015.1007471 PMC4536939

